# Risk of infection during treatment with oral Janus kinase inhibitors in alopecia areata: A systematic review and meta-analysis

**DOI:** 10.1016/j.jdin.2026.09.015

**Published:** 2026-10-06

**Authors:** Dian-Bin Huang, Li-Wen Chiu, Wei-Cheng Fang

**Affiliations:** a Department of General Medicine, Kaohsiung Medical University Hospital, Kaohsiung, Taiwan; b Department of Dermatology, Kaohsiung Medical University Hospital, Kaohsiung, Taiwan; c Department of Dermatology, College of Medicine, Kaohsiung Medical University, Kaohsiung, Taiwan; d Department of Dermatology, Kaohsiung Municipal Siaogang Hospital, Kaohsiung, Taiwan

**Keywords:** alopecia areata, infection, JAK inhibitors, Janus kinase inhibitors, meta-analysis, systematic review

*To the Editor:* Oral Janus kinase inhibitors (JAKi) have become important systemic treatment options for alopecia areata (AA), and baricitinib, ritlecitinib, and deuruxolitinib are currently approved by the Food and Drug Administration for severe AA.^1^ Previous meta-analyses have reported significantly increased risks of selected infections with JAKi, yet most pooled AA with other dermatologic indications.^2^, ^3^, ^4^ Consequently, infection risks with oral JAKi in AA remain incompletely characterized. To address this gap, we conducted a systematic review and meta-analysis of randomized controlled trials (RCTs) to provide an AA-specific assessment of infection risks during oral JAKi treatment.

Our study was registered in International Prospective Register of Systematic Reviews (PROSPERO) (CRD420261415727) and reported in accordance with the Preferred Reporting Items for Systematic Reviews and Meta-Analyses guidelines (Supplementary Table I, available via Mendeley at https://data.mendeley.com/datasets/nptjf2872n/1). We searched PubMed, Embase, Cochrane Central Register of Controlled Trials (CENTRAL), Web of Science, and ClinicalTrials.gov from inception to June 6, 2026 (Supplementary Table II, available via Mendeley at https://data.mendeley.com/datasets/nptjf2872n/1). Eligible studies were blinded, placebo-controlled, phase 2 or 3 RCTs investigating oral JAKi in patients with AA and reporting quantitative data on infection outcomes. Random-effects meta-analyses were performed to pool risk ratios with 95% confidence intervals using safety populations. Sensitivity analyses using the leave-one-out method and subgroup analyses stratified by individual oral JAKi were conducted. Post hoc exploratory sensitivity analyses using the Peto odds ratio method were performed to assess robustness to sparse-event handling. Detailed methodology is provided in the Supplementary Materials, available via Mendeley at https://data.mendeley.com/datasets/nptjf2872n/1.

A total of 12 RCTs enrolling 4131 participants (mean age: 36.8 years) were included (Supplementary Fig 1 and Table III, available via Mendeley at https://data.mendeley.com/datasets/nptjf2872n/1). Oral JAKi were not associated with statistically significant increases in the risks of serious infections, herpes zoster, herpes simplex, influenza, COVID-19, nasopharyngitis, upper respiratory tract infection, or urinary tract infection in AA (Table I and Supplementary Figs 2 to 9, available via Mendeley at https://data.mendeley.com/datasets/nptjf2872n/1). No opportunistic infections were reported across the included studies. Folliculitis was associated with an increased risk (risk ratio: 2.24, 95% CI: 1.19-4.23, *P* = .013) (Fig 1); however, this finding should be considered exploratory because no adjustment for multiplicity was performed. Post hoc Peto odds ratio analyses showed nominally significant increases in the risks of folliculitis, herpes zoster, nasopharyngitis, and urinary tract infection (Supplementary Table IX, available via Mendeley at https://data.mendeley.com/datasets/nptjf2872n/1). Detailed results are provided in the Supplementary Materials, available via Mendeley at https://data.mendeley.com/datasets/nptjf2872n/1.

**Table I:** Summary of meta-analyses of prespecified infections during treatment with oral Janus kinase inhibitors compared with placebo in patients with alopecia areata

Outcome	No. of studies	JAKi, *n*/*N*	Placebo, *n*/*N*	RR (95% CI)	*P* value	*I*^2^ (%)
Folliculitis	7	78/1520	10/517	2.242 (1.188-4.232)	.013	0.00
Serious infections	9	17/2473	2/811	1.230 (0.464-3.260)	.677	0.00
Herpes zoster	8	27/2722	2/913	1.992 (0.761-5.216)	.160	0.00
Herpes simplex	5	24/1353	16/532	0.557 (0.295-1.052)	.071	0.00
Influenza	4	6/785	3/243	0.797 (0.098-6.486)	.832	48.26
COVID-19	5	160/1269	49/446	1.191 (0.884-1.606)	.250	0.00
Nasopharyngitis	9	233/2682	56/901	1.253 (0.924-1.697)	.146	6.68
Upper respiratory tract infection	12	227/3026	77/1063	1.042 (0.811-1.337)	.749	0.00
Urinary tract infection	9	80/2774	16/944	1.565 (0.899-2.722)	.113	0.00

For each infection outcome, active treatment arms were combined into a single Janus kinase inhibitor group in multi-arm studies. *n* denotes the number of events, and *N* denotes the safety population denominator from studies contributing data to each outcome.

*CI*, Confidence interval; *JAKi*, Janus kinase inhibitor; *RR*, risk ratio.

**Fig 1:**
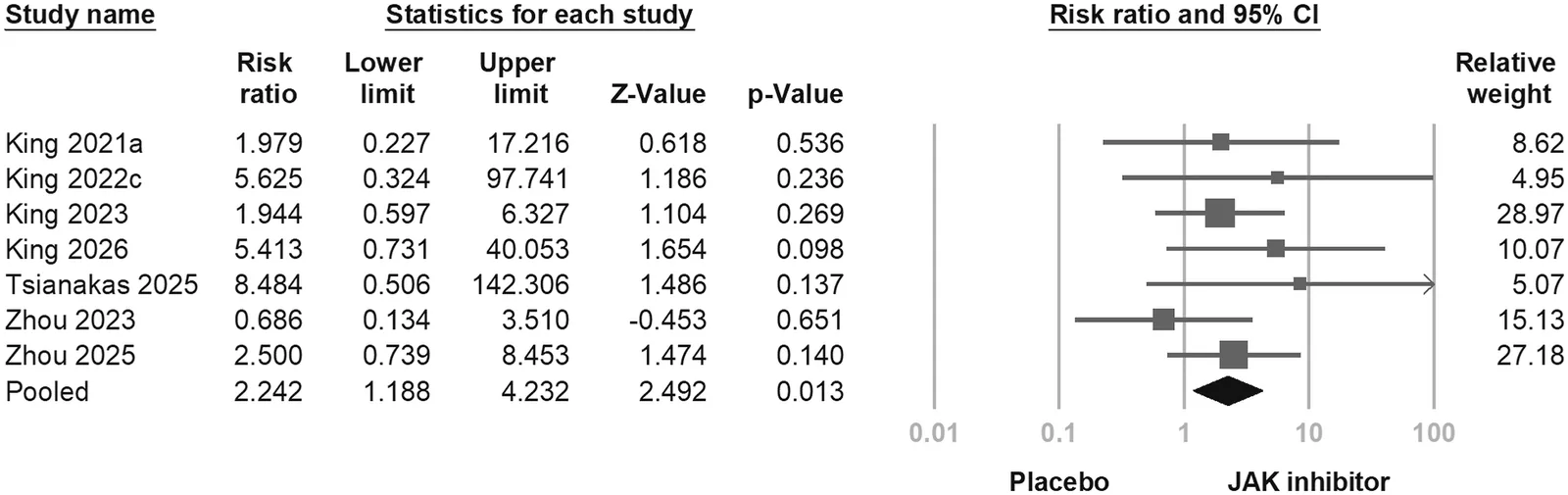
Forest plot of risk ratios for folliculitis during treatment with oral Janus kinase inhibitors compared with placebo. *Squares* indicate study-specific risk ratios, with square size proportional to relative weight. *Horizontal lines* indicate 95% confidence intervals. The *diamond* indicates the pooled estimate. *CI*, Confidence interval; *JAK*, Janus kinase.

Overall, this meta-analysis yielded imprecise short-term estimates of infection risk while identifying an exploratory association between oral JAKi and an increased risk of folliculitis in AA. These findings suggest folliculitis as a cutaneous adverse event warranting monitoring, while clinically important increases in the risks of serious infections, herpes zoster, and urinary tract infection could not be excluded.

Limitations include short follow-up periods in the included RCTs, relatively young and clinically selected study populations, sparse and zero-event data for rare infections, exclusion of studies with zero events in both groups from relative-effect meta-analyses, sensitivity of some estimates to analytic methods, incomplete reporting for some infection outcomes, and lack of dose-response assessment. Long-term studies with larger populations are warranted to clarify rare or delayed infection risks among patients with AA.

### Declaration of generative AI and AI-assisted technologies in the writing process

During the preparation of this work the authors used ChatGPT (OpenAI) in order to improve clarity and readability. After using this tool, the authors reviewed and edited the content as needed and take full responsibility for the content of the published article.

## Conflicts of interest

None disclosed.
